# Temporal complexity measure of reaction time series: Operational versus event time

**DOI:** 10.1002/brb3.3069

**Published:** 2023-05-23

**Authors:** Korosh Mahmoodi, Scott E. Kerick, Paolo Grigolini, Piotr J. Franaszczuk, Bruce J. West

**Affiliations:** ^1^ US Combat Capabilities Command Army Research Laboratory, Aberdeen Proving Ground Aberdeen Maryland USA; ^2^ Center for Nonlinear Science University of North Texas Denton Texas USA; ^3^ Department of Neurology Johns Hopkins University School of Medicine Baltimore Maryland USA; ^4^ Office of Research and Innovation North Carolina State University Raleigh North Carolina USA

**Keywords:** detrended fluctuation analysis, reaction time series, temporal complexity, time‐stress

## Abstract

**Introduction:**

Detrended fluctuation analysis (DFA) is a well‐established method to evaluate scaling indices of time series, which categorize the dynamics of complex systems. In the literature, DFA has been used to study the fluctuations of reaction time *Y*(*n*) time series, where *n* is the trial number.

**Methods:**

Herein we propose treating each reaction time as a duration time that changes the representation from operational (trial number) time *n* to event (temporal) time *t*, or *X*(*t*). The DFA algorithm was then applied to the *X*(*t*) time series to evaluate scaling indices. The dataset analyzed is based on a Go–NoGo shooting task that was performed by 30 participants under low and high time‐stress conditions in each of six repeated sessions over a 3‐week period.

**Results:**

This new perspective leads to quantitatively better results in (1) differentiating scaling indices between low versus high time‐stress conditions and (2) predicting task performance outcomes.

**Conclusion:**

We show that by changing from operational time to event time, the DFA allows discrimination of time‐stress conditions and predicts performance outcomes.

## Graphical abstract

We introduce a novel conceptual and analytical framework for estimating the complexity of short behavioral time series using Detrended Fluctuation Analysis (DFA). Analyzing the dataset based on a Go‐NoGo shooting task by this method, we found a clear classification of complexity indices between Low and High time‐stress conditions. We also found clear relations between complexity indices and errors of commission.

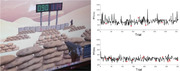

## 1 INTRODUCTION

Reaction time has been the standard measure of perceptual‐motor and cognitive performance for more than a century (Luce, 1986; Meyer et al., 1988). Typically, reaction time data have been analyzed based on statistical moments of the distribution of reaction time latencies over all trials in various experimental conditions (mean, variance, skewness, and kurtosis). However, this approach fails to capture the underlying temporal dynamics of the intermittent fluctuations inherent in ordered sequences of reaction times over trials, which are central to modeling neural latency mechanisms at a macroscopic scale (Luce, 1986; Smith & Ratcliff, 2004). When analyzed as temporal sequences of ordered reaction time trials, that is, a time series of reaction time latencies from 1 to N trials (hereafter referred to as *Y*(*n*)), previous research reveals inverse power law (IPL) statistics in cognitive and behavioral time series data (Correll, 2008; Gilden, 2001, 1995; Grigolini et al., 2009; Kello et al., 2007, 2010; Simola et al., 2017; Wijnants et al., 2009). Such IPL temporal probability density functions (PDFs) are known to have IPL power spectral densities (PSD) *S*(*f*) $\;\propto f^{-\beta }$ where *f* is the frequency, and this 1/*f*‐variability has been interpreted to be a consequence of long‐range temporal correlations (LRTCs). The PSD IPL index β is related to the IPL index *α* of the temporal PDF measured by detrended fluctuation analysis (DFA) (2*α*−1 = β) and determines the well‐known 1/*f‐noise* when β=1. Simola et al. (2017) argued that the LRTC arises from critical dynamics and use this perspective to resolve a long‐standing controversy concerning the source of LRTC in response time fluctuations. They concluded: “Our findings thus favor the hypothesis that LRTCs are caused [by] the system being in a critical state over the idea that these LRTCs would reflect long‐memory dynamics” (p. 2).

### 1.1 Introducing crucial events (CEs)

Researchers have shown that individuals performing more difficult tasks requiring greater cognitive effort exhibit relatively smaller IPL indices β of reaction time series, *Y*(*n*), than those observed for simpler tasks. For example, in a series of experiments, Kello et al. (2007) found smaller IPL indices β for variable versus fixed trial intervals, random versus patterned cues, and unpreviewed versus previewed trials. Wijnants et al. (2009) showed that IPL scaling β indices become greater (more clearly patterned 1/*f*‐variability) as participants become more skilled in a precision‐aiming motor task over blocks of practice. Correll (2008) found smaller IPL indices β in participants who reported a high level of effort to avoid racial bias while responding to black versus white target stimuli under threat/no‐threat conditions (see also Grigolini et al., 2009 for a theoretical explanation). Overall, the evidence suggests that greater IPL indices β are indicative of greater levels of cognitive and behavioral performance and/or performance of simpler tasks.

In a similar Go–NoGo paradigm, Simola et al. (2017) showed that IPL scaling indices β were inversely related to cognitive flexibility, as measured by errors of commission (i.e., by greater IPL indices β associated with a lower number of errors). Although Simola et al. (2017) found support for criticality being the basis of LRTCs, they do not consider the alternative explanation that CEs might offer in a self‐organizing critical system. CEs are defined to be a sequence of events separated by time intervals that are statistically independent of one another. The time intervals between independent events are generated by an IPL PDF with the IPL index μ that lies in the interval 1<μ<3. The relationship between complexity indices *α*, *β*, and μ is summarized in Table 1.

**TABLE 1:** The relation among the scaling indices α,β,andμ, which can be measured, respectively, from detrended fluctuation analysis (DFA), power spectral densities (PSD) *S*(*f*), and the waiting‐time probability density function (PDF) *ψ*(*t*) of the time series

Scaled functions		Parameter relations	Parameter range
Waiting‐time PDF	*ψ*(*t*) ∝ *t* ^−^ *^μ^*	*μ* = 3 − *β*	1 < *μ* < 3
Power spectrum	*S*(*f*) ∝ *f* ^−^ *^β^*		
Scale variable	*X*(*t*) ∝ *t^α^*	*μ* = 4 − *2α*	

*Note*: The value *μ* = 2 is the boundary between the underlying process having a finite (*μ >* 2) or an infinite (*μ <* 2) average waiting time and is also the point at which *β* = 1 where the process is that of 1/*f*‐noise.

Finally, the statistical distribution of the CEs is renewal and consequently possesses a new kind of memory, one associated with renewal processes. This new kind of memory was discovered by Allegrini et al. (2002) and was given the apt name “memory beyond memory.” It is this new kind of memory that is often confused with LRTC (West & Grigolini, 2021), the latter reflecting long‐memory dynamics, whereas the former is a statistical property of the renewal CEs. The leading question therefore arises as to whether the true source of the IPL PDF of the *Y*(*n*) can be quantitatively determined. If the *Y*(*n*) time series is renewal, which is to say it is a CE time series, the above question could be answered because the time series would necessarily have the following properties:

“The time series generated by complex processes are characterized by three regimes: the short‐time regime, where the true complexity of the process is not yet perceived; an intermediate‐time regime driven by the CEs; and a long‐time regime where the process can be mistaken for an ordinary statistical process” (West & Grigolini, 2021, p. 16).

### 1.2 Empirical data

**FIGURE 1:**
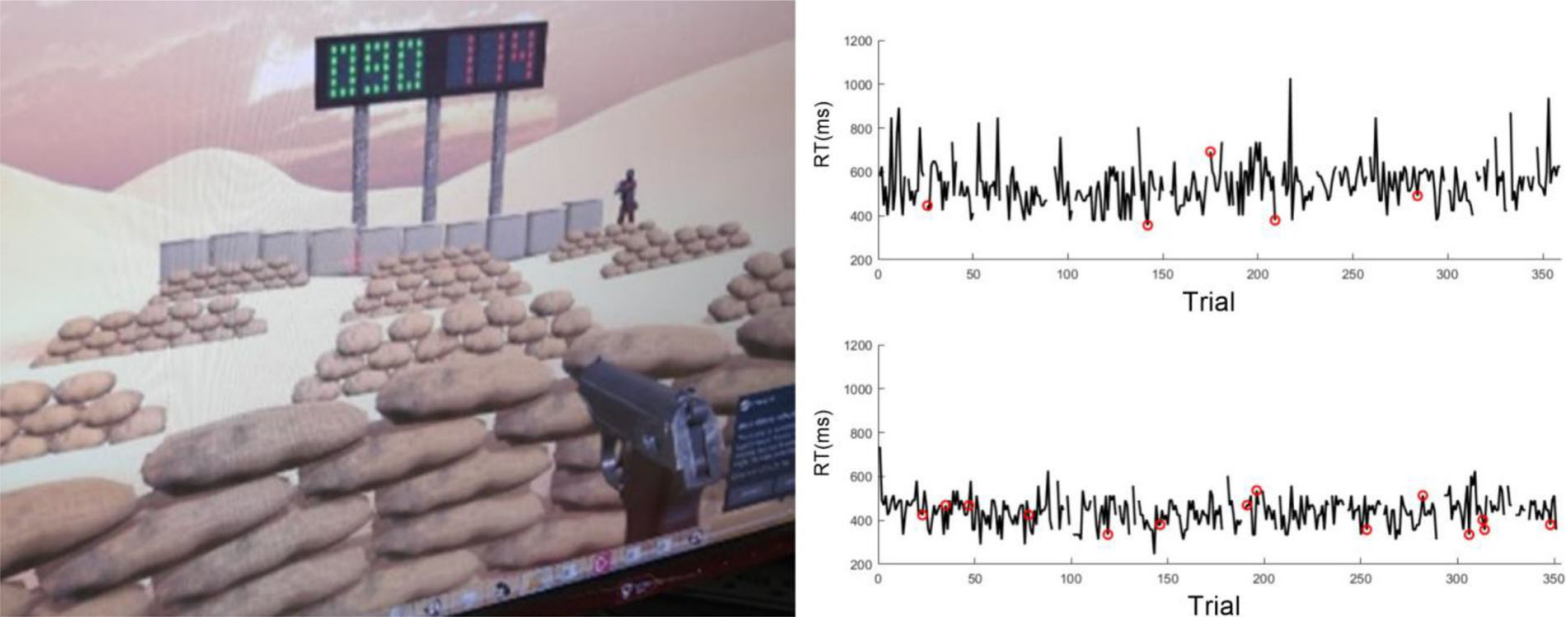
Left panel: Virtual reality Go–NoGo shooting task via participant's first‐person perspective in HTC Vive; right panel: example participant's reaction time trial series in low (top) and high (bottom) time‐stress conditions. Red circles indicate errors of commission; gaps in time series indicate lack of trigger responses on those trials (i.e., correct omissions to friendly targets or errors of omission to enemy targets).

In the present work, the existence of IPL scaling indices is examined using data previously recorded during a Go–NoGo shooting task under low and high time‐stress conditions as part of a neurofeedback training study (See Task and Procedures and Figure 1 below, and Kerick et al., 2023 for more details). We are not aware of previous research that has investigated the effects of time stress on scaling indices over different time scales within and across repeated experimental sessions spanning several days or weeks. In this research, we define short‐time regimes at the single‐trial level (milliseconds), intermediate‐time regimes at the single‐task level within each session (each low and high time‐stress conditions; 8–10 min), and long‐time regimes at the between‐session level (6 sessions over a 3‐week period). Herein, we examine the effects of time stress on IPL scaling indices under conditions with shorter (high stress; ∼250–750 ms) and longer (low stress; ∼600–1000 ms) time allocated for the task (i.e., target exposure durations) within and across six sessions.

The shooting task required the participants to scan the environment for targets appearing over spatially distributed locations, detect and orient the weapon toward the target upon its appearance, identify whether the target is friend or foe, decide whether to shoot or not, and if the decision is to shoot, the participant must accurately aim and fire the weapon within the constrained time interval in which the target is exposed. As a task designed to assess inhibitory executive control mechanisms by evaluating errors of commission (shots fired at friendly targets), this shooting task variant represents a more ecologically valid, complex, and challenging Go–NoGo task than has been implemented in previous research. Typically, Go–NoGo tasks consist of stimuli appearing on a computer screen in a fixed location, and Go responses are simple button presses on a keyboard (Correll, 2008; Simola et al., 2017). We also have a relatively small number of trials in each condition in each session (*n* = 360) because the previous study was structured to limit the overall length of the experiment, which could introduce additional effects of boredom or fatigue. Thus, these data present a challenge for rigorous testing of theoretical models and analytical approaches for identifying power laws, IPLs, and scaling from shorter time series generated under more complicated task conditions than in previous applications.

Because of the comparatively small dataset resulting from our relatively short reaction time series, the previously established methods do not provide reasonable estimates of the IPL scaling index β. The scaling index in previous studies is typically estimated by finding the slope of a linear approximation to the PSD function in log–log coordinates. The PSD estimate is usually achieved by applying the fast Fourier transform to the reaction time trial series (1:*N*), which requires a relatively large number of stationary data points to obtain low variance and stable PSD estimates. Typically, researchers implement tasks consisting of 1000 or more trials to enable stable spectral estimates, and the evolution of longer periods to observe slow fluctuations, although as few as 200 trials have been employed (Correll, 2008). Because the IPL selection of interest in a PSD occurs at low frequency, we need to have long time series to obtain meaningful IPL behavior for at least one decade of frequency. As such, short time series create noisy PSD estimates, and consequently IPLs of uncertain slope, with a corresponding IPL index β.

### 1.3 New way to process reaction time series

DFA has been widely used to measure the scaling indices *α* of reaction time series (Delignieres et al., 2006; Simola et al., 2017; Wijnants et al., 2009). In previous studies, the signals analyzed by DFA have been the *Y*(*n*) time series measured at each trial *n* of the experiments. This represents a time series expressed in trial intervals referred to as operational time (Turalska & West, 2018). For simple tasks, IPL relations have been found using the IPL index for the PSD β, or power law relations using DFA *α* (Correll, 2008; Gilden, 2001; Kello et al., 2007; Simola et al., 2017). However, we show herein that for reaction time series from a more realistic task having short trial sequences, neither the PSD method nor the DFA technique is able to extract a reliable measure of the scaling indices of the process.

**FIGURE 2:**
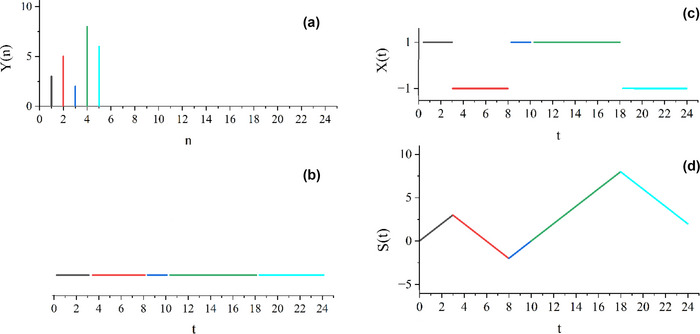
The schematics of changing data from operational time *Y*(*n*) (a) to event time *X*(*t*) (c): (Panel a) the initial *Y*(*n*) data; (Panel b) each data point in operational time *Y*(*n*) is considered a duration time; (Panel c) to create *X*(*t*), each duration is filled with +1 or −1, assigned randomly (in this example, each latency interval is filled with +1, −1, +1, +1, and −1, respectively); (Panel d) the S(*t*) trajectory is the cumulative sum of *X*(*t*), notice that because of the random assignment of signs, there are many possible trajectories in event time *X*(*t*)’s. Thus, we use an ensemble average for our analysis to consider this variety, especially for short time series. In panels (b) and (c), *t* represents accumulated durations of reaction times. Note that the trajectory *X*(*t*) hosts both CEs and non‐CEs from the empirical trials. The trajectory is then analyzed using detrended fluctuation analysis (DFA) to determine the scaling index *α*, which is not the same index as that obtained by applying DFA to *Y*(*n*). This difference is discussed in the following section.

Herein, we propose a new perspective by considering each reaction time as a time interval (*t* in ms) rather than a trial number (*n*) and filling each reaction time latency interval (i.e., event time) with the noise of fixed amplitude (of magnitude 1 and a random chosen sign ± for the interval; see Figure 2). This represents a latency or duration time series expressed as the new variable *X*(*t*), which is a function of temporal intervals (not trial numbers) referred to as chronological time, see Section 2.5 for details (Turalska & West, 2018).^1^ This secondary time series, *X*(*t*) (Figure 2, panel c) represents the rigidity (constant noise) and flexibility (changing sign) of the process. By measuring the complexity of the *X*(*t*) time series, using DFA, we find a clear classification of scaling indices between low and high time‐stress conditions. We also find clear trends between scaling indices and errors of commission. Neither of these findings was observed for the traditional DFA analysis of *Y*(*n*) time series.

To show the connection of the scaling index *α* of the *X*(*t*) time series, measured using DFA, to the temporal complexity index *μ*, we introduce a simulation where the relation between the two scaling indices can be established and tested. The simulated *Y*(*n*) time series, with an IPL PDF having an IPL index *μ*, is generated using the well‐known Manneville map (Manneville, 1980). Then, we created the corresponding simulated *X*(*t*) time series from the simulated *Y*(*n*) and measured the scaling index *α* via DFA. Consequently, we find the empirical relation between *α* and μ,μ=4−2α, making it possible to connect *α* to the IPL index of the PSD (*β* = 3 − *μ*) and therefore adapt the technique to empirical reaction time datasets.

## 2 METHODS

### 2.1 Participants

Thirty (13 female) young healthy adults (ages 18–40 years; mean 24.99 ± 3.21 years) participated in 6 separate sessions within a 3‐week interval. Volunteers who agreed to participate were asked to read and sign an Informed Consent Agreement (approved by the Human Use Committee at the US Army Research Laboratory and the Institutional Review Board at the University of Maryland, Baltimore County, in accordance with the Declaration of Helsinki and the U.S. Code of Federal Regulations).

### 2.2 Task design

The Go–NoGo task was implemented in virtual reality using HTC Vive (https://www.vive.com/us/). In each session, the participants completed four blocks of 90 trials (360 total trials) in each low and high time‐stress condition (2160 total trials in each condition over 6 sessions). The durations of each shooting task condition in each session were approximately 8–10 min. Pop‐up targets were pseudorandomly distributed 40 times at each of 9 range locations (three simulated distances (near, mid, and far) by three lanes (left, center, and right) and exposed at variable onset intervals (1000 ± 500 ms over a Gaussian‐distributed range of 100 ms increments) for various target exposure durations (see Figure 1). The probability of targets (enemy; red) to nontargets (friendly; green) was 0.90/0.10, respectively (i.e., 324 enemy targets and 36 friendly targets were presented) to induce a prepotent response bias (see Kerick et al., 2023 for more details).

The participants were instructed to “shoot enemy targets as quickly and accurately as possible, while refraining from shooting at friendly targets.” Time‐stress conditions were individualized based on a pretesting performance thresholding procedure to account for individual differences in participants’ ability to perform the task. This was done by empirically determining target exposure durations corresponding to the 50th (High time‐stress) and 90th (Low time‐stress) percentile hit‐rates in response to 100 enemy targets using psychophysical methods (method of limits; Ehrenstein & Ehrenstein, 1999). The mean (SD) target exposure time in the low time‐stress condition was 842.78 ms (216.65 ms) and 547.96 ms (223.20 ms) in the high. Figure 1 provides example reaction time series from a representative subject in low and high time‐stress conditions in one session. Herein we analyze the scaling index of reaction times series and examine their relation to the performance measure errors of commission, defined as shots fired at friendly targets (failure to inhibit incorrect responses).

### 2.3 Data analysis

Across all subjects, sessions, and conditions, 122,153 raw reaction time trials were first pruned of extreme latency values (100 ms < reaction time < 1500 ms). This resulted in the removal of 659 reaction time trials (121,494 preserved for subsequent analysis). After trimming the datasets of outliers, we proceeded with DFA analyses of *Y*(*n*) and *X*(*t*), each at the single session time scale (360 trials) and across all 6 sessions concatenated (2160 trials). Concatenating trials from all six sessions enabled analyses of longer time series over longer time scales. We also simulated *Y*(*n*) and *X*(*t*) time series consisting of temporal complexity consistent with our empirical observations at each time scale. It should be noted here that the number of reaction time trials for each subject, condition, and session was variable because variable numbers of errors of commission (friendly‐fire errors) and omission (failure to fire at enemy targets) were inherent in the data. The mean (SD) reaction time latencies in the Low time‐stress condition were 529.00 ms (164.79 ms) and 441.44 ms (121.57 ms) in the high time‐stress condition collapsed across sessions and was a statistically significant main effect of condition (*p* < .01) (see Kerick et al., 2023 for additional analyses).

### 2.4 DFA analysis of *Y*(*n*) time series

For each subject under each condition and time scale, we computed the percentage of errors of commission and applied DFA analyses to *Y*(*n*) time series. DFA is applied to reveal long‐range correlations in time series (Peng et al., 1995). Here we explain the steps for DFA on *Y*(*n*): (1) integrating *Y*(*n*) over *n* trails to obtainS(n); (2) dividing S(n)into windows of length *w*; (3) deriving least squares line to fit the dataset in each window, $S_{w}\left(n\right);\;$(4) calculating the root mean square amplitude in each window *w*, *F*(*w*) of Equation (1); (5) average the *F*(*w*)s; (6) repeat 1 through 5 for different *w*s, and (7) evaluate the scaling *α* as the slope of *F*(*w*) versus *w* in a log‐log plot:

(1) $$F\left(w\right)=\;\sqrt{\frac{1}{N}\sum_{n\;=\;1}^{N}{\left(S\left(n\right)-S_{w}\left(n\right)\right)}^{2}}$$

### 2.5 DFA analysis of *X*(*t*) time series

Figure 2 shows a schematic for transforming data from operational time (*Y*(*n*)) to event time (*X*(*t*)). Panel a of this figure shows some hypothetical data points in trial time *n*, *Y*(*n*). In panel b, each of these data points is transformed into a latency interval corresponding to the reaction times in panel a, and in panel c, these latency intervals are filled with +1 or −1, chosen randomly, to create *X*(*t*). It is important to note here that time intervals between trials (i.e., periods following responses and preceding the next target onset) are disregarded. Thus, *X*(*t*) does not reflect continuous time, but rather the concatenation of discrete reaction time event times. The signal in panel c is then subjected to DFA. The steps for DFA on *X*(*t*) are the same as explained for DFA on *Y*(*n*) (replacing *n* with *t*). Panel d shows the cumulative sum of the input data in panel c (S(t)) as required in step (1) of the DFA. For real datasets of reaction times, the reaction times were first rounded to three significant digits (in ms), and the resulting *X*(*t*) time series (Figure 2, panel c) was processed and using DFA the corresponding scaling index *α* calculated. Notice that such a generated *X*(*t*) time series has a random component (i.e., the sign of each duration time), so, we can make different *X*(*t*) time series. To consider many possibilities, for each *Y*(*n*) time series, we ran DFA over 100 generated *X*(*t*) time series and evaluated the average value of *α*.

### 2.6 Comparing the DFA of *Y*(*n*) and *X*(*t*)

**FIGURE 3:**
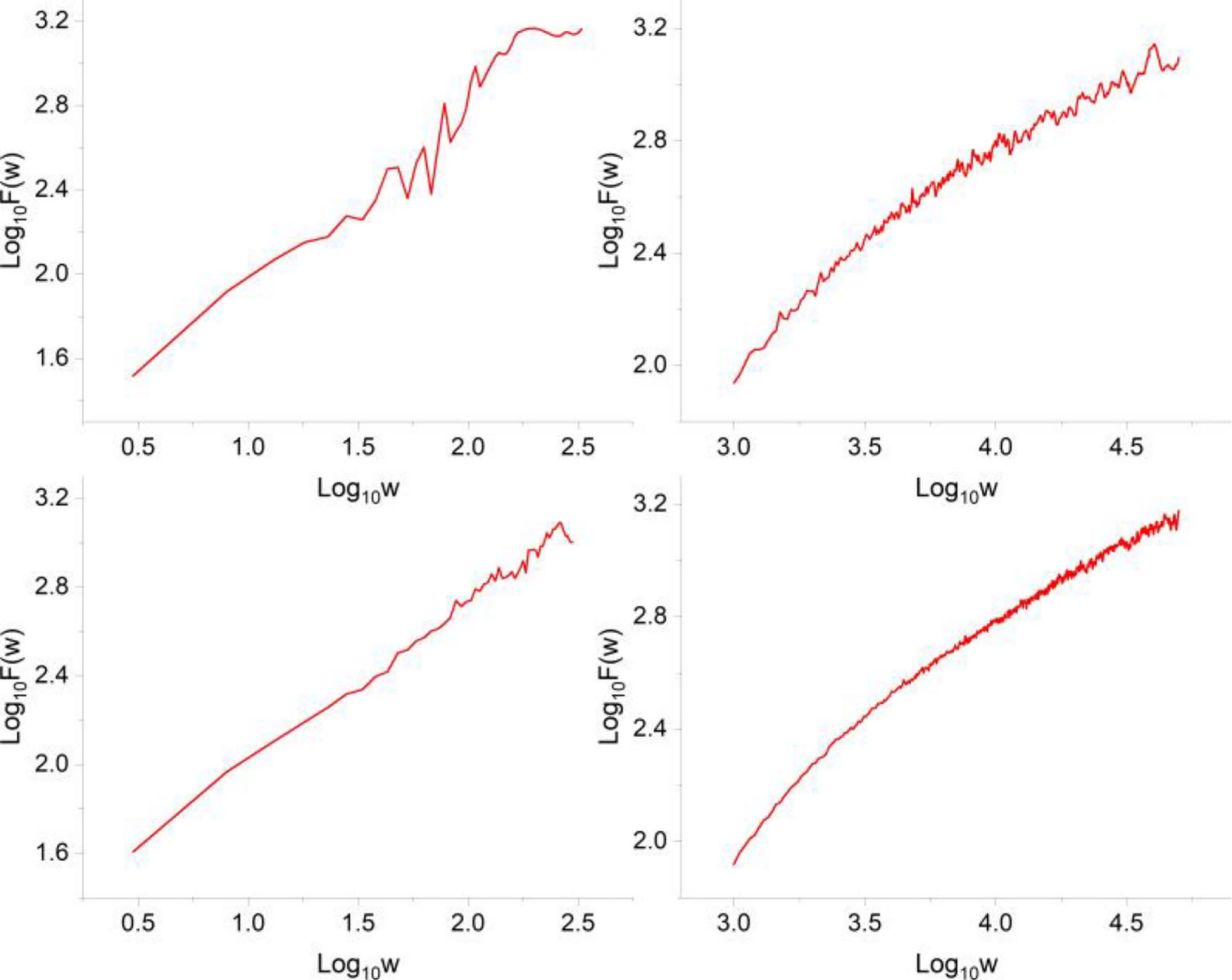
The left panels show the graph for detrended fluctuation analysis (DFA) on the *Y*(*n*) time series of one participant during an individual session (top–left) and during all sessions appended (bottom–left). The right panels show the graph for DFA on the corresponding *X*(*t*) time series (created according to the description in Figure 2) of the same participant during an individual session (top–right) and during all sessions appended (bottom–right).

Figure 3 shows the differences in the DFA graph when *Y*(*n*) time series of an individual was used (left panels) versus the DFA graph on its corresponding *X*(*t*) (right panels). The *Y*(*n*) were taken from a representative participant in one session (top panels) and across all six sessions appended (bottom panels), in the low time‐stress condition. As can be seen in the figure, the DFA graph of the *X*(*t*) has fewer fluctuations and a more extended power–law domain.

### 2.7 DFA on simulated reaction times with different temporal complexity *μ*

To introduce the method used herein, and the relating temporal complexity to scaling measured by DFA, we used simulated data, temporally complex, using turbulence intermittency as prescribed by MannevillleManneville (1980). To make the numerical generation of the events faster, we adopted an idealized version of this map in the following way (Buiatti et al., 1999): Each reaction time can be calculated by transforming the sequence *y_i_* of random numbers uniformly distributed in the interval (0, 1) into the temporal sequence *τ_i_*:

(2) $$\tau_{i}=T\frac{1}{y_{i}^{\left(\frac{1}{\mu -1}\right)}-1}$$

where *μ* (*μ* > 1) is the temporal scaling index of the generated time series. The *τ_s_* are generated by a hyperbolic PDF *ψ*(*τ*) defined by

(3) $$\psi \;\left(\tau \right)=\;\left(\mu -1\right)\frac{T^{\left(\mu -1\right)}}{{\left(\tau +T\right)}^{\mu }}$$

which is properly normalized. The time constant *T* defines the time necessary to turn microscopic dynamics into a process with an evident IPL index *μ*. For 1 < *μ* < 3, this PDF represents a complex system, which is a renewal process of CEs. For *μ* > 3, the dynamics falls into the region of normal statistics (Annunziato & Grigolini, 2000) and is no longer complex. We take the set of *τ_i_* generated by the Manneville map, with a given scaling index *μ*, as the simulated reaction times *Y*(*n*) and create the corresponding *X*(*t*) time series. The DFA was then used to process the resulting *X*(*t*) time series generated using the prescription in Figure 2.

**FIGURE 4:**
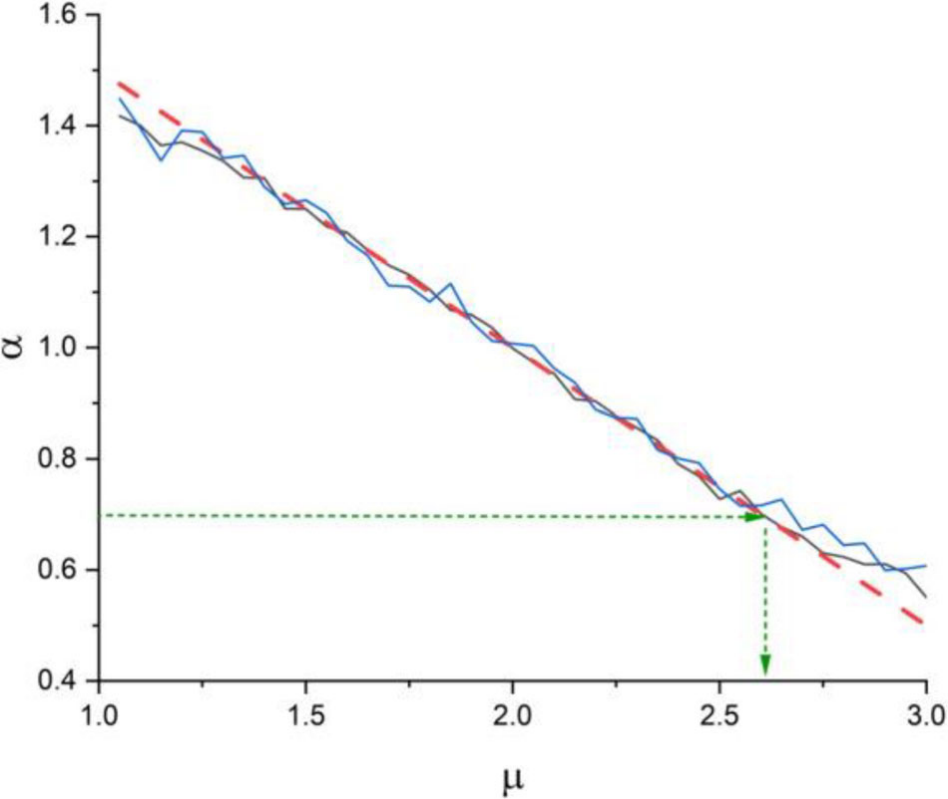
The detrended fluctuation analysis (DFA) of simulated reaction time series. Each *Y*(*n*) is a simulated duration time generated by the idealized Manneville map (Buiatti et al., 1999) with corresponding temporal complexity index *μ*. The simulated *Y*(*n*) time series used to generate the corresponding simulated *X*(*t*) time series using the description of Figure 2. The length of the simulated trajectories (*X*(*t*) time series) were chosen to be similar to the *X*(*t*) dataset in the session level (blue line) and the appended sessions (black line). The dotted red line is the linear fit (Equation 4). The dotted green lines show an example of estimating the temporal complexity index *μ* from measured *α* using DFA applied to the *X*(*t*) time series.

Figure 4 shows the resulting relationship between the temporal complexity index *μ* of the simulated time series and the scaling index *α* evaluated using DFA analysis of the *X*(*t*) time series. The linear fit can be approximated using

(4) μ=4−2α

So, this method is an indirect way of evaluating the temporal complexity index *μ* of short time series.

### 2.8 Relation between *α* and 1/*f‐*variability

Here we connect the scaling index *α* measured via DFA processing of the *X*(*t*) time series to the power–law index of $\;f^{-\beta }\;$variability. It is known that the series of events created by the Manneville map with scaling index *μ* has an IPL PDF (Lukovic & Grigolini, 2008):

(5) $$S\left(f\right)\propto f^{-\beta }\propto f^{-\left(3-\mu \right)}.$$

Substituting the relation between index given by Equation (4) into this expression, we obtain

(6) $$S\left(f\right)\propto f^{-\left(2\alpha -1\right)}.$$

So, only *α* = 1 corresponds to a perfect 1/*f‐*noise.

### 2.9 Statistical analysis

Wilcoxon matched‐pairs signed rank tests were applied to determine whether errors of commission and values of the scaling index for the two DFA analyses differed between low and high time‐stress conditions at each time scale. Separate linear regression analyses were also applied using values of the scaling index (DFA processing of *Y*(*n*) and *X*(*t*)) as predictors and errors of commission as response outcomes under each condition and time scale to determine whether the strength of the predictors differed between conditions.

## 3 RESULTS

### 3.1 Differences between time‐stress conditions for errors of commission and DFA scaling indices

**FIGURE 5:**
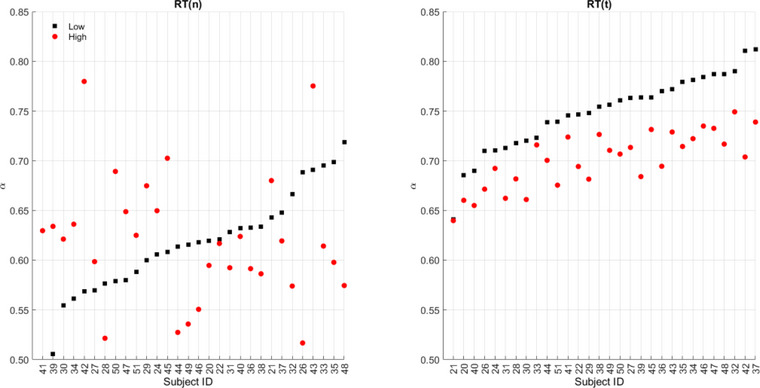
Left panel: scaling indices measured by detrended fluctuation analysis (DFA) analysis of the *Y*(*n*) time series of participants in high (red dots) and low time‐stress (black squares) conditions; right panel: scaling indices measured by DFA analysis of the *X*(*t*) time series of each participant in high (red dots) and low time‐stress (black squares) conditions. Paired data are sorted by indices in low time‐stress condition in both panels. Each data point is averaged over 100 DFA analyses of the *X*(*t*) time series. Note the dispersion of red dots around black squares in the left panel (*Y*(*n*)) versus the consistently lower red dots around black squares in the right panel (*X*(*t*)), highlighting the superiority of the latter in differentiating scaling indices in the low versus high time‐stress conditions.

Wilcoxon matched‐pairs signed rank tests applied to errors of commission and DFA scaling indices of *Y*(*n*) and *X*(*t*) time series for individual sessions revealed statistically significant differences for errors of commission (*Z* = −4.5560; *p* < .001) and *X*(*t*) (*Z* = 4.7821; *p* < .001), but not for *Y*(*n*) (*Z* = −0.1954; *p* = .85). For all sessions appended, statistically significant differences were observed for errors of commission (*Z* = −9.3265; *p* < .001) and *X*(*t*) (*Z* = 11.0996; *p* < .001), but not for *Y*(*n*) (*Z* = −0.3871; *p* = .70). Table 2 provides median and quantile (0.25 0.75) values for each dependent variable under each time‐stress condition. Figure 5 shows differences of scaling indices between low and high time‐stress conditions for the DFA analysis of *Y*(*n*) and *X*(*t*) time series. The DFA analysis of the *X*(*t*) time series was able to categorize the scaling indices of the two conditions, whereas *Y*(*n*) was not.

**TABLE 2:** Median and quantile (0.25 0.75) values by time‐stress condition

Variable	Low	High
Individual sessions		
Errors of commission	0.17 (0.11 0.22)	0.33 (0.24 0.48)
α for *Y*(*n*)	0.62 (0.58 0.64)	0.62 (0.59 0.65)
α for *X*(*t*)	0.75 (0.72 0.78)	0.71 (0.68 0.72)
All sessions appended		
Errors of commission	0.15 (0.08 0.28)	0.33 (0.19 0.50)
α for *Y*(*n*)	0.59 (0.51 0.65)	0.60 (0.52 0.66)
α for *X*(*t*)	0.75 (0.71 0.77)	0.69 (0.67 0.72)

**FIGURE 6:**
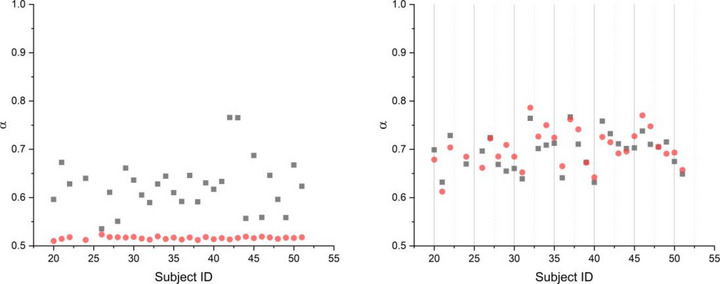
The left panel shows the results of applying detrended fluctuation analysis (DFA) to the *Y*(*n*) (black squares) and to shuffled *Y*(*n*) (red dots). The right panel depicts the results of applying DFA to *X*(*t*) created from *Y*(*n*) (black squares) and to *X*(*t*) created form the shuffled *Y*(*n*) (red dots).

Next, we evaluated whether shuffling the reaction time series, *Y*(*n*), prior to DFA analysis destroys the long‐term correlations inherent in the intact reaction time series and lowers scaling indices. Figure 6 compares the DFA applied to randomly shuffled *Y*(*n*) and their corresponding *X*(*t*) time series. After shuffling, the DFA analysis of *Y*(*n*) time series yielded scaling indices close to *α* = .5 (randomness), whereas in the case of DFA analysis of the *X*(*t*) time series, created from the shuffled *Y*(*n*), does not significantly change the scaling indices.

### 3.2 Regression analyses of DFA scaling indices for predicting errors of commission

Regression analyses revealed that scaling indices derived from DFA analysis of *Y*(*n*) time series was not a significant predictor of errors of commission in either low or high time‐stress conditions for either individual sessions or all sessions appended (all *p* values >.05; see Table 3). However, scaling indices derived from the DFA analysis of the *X*(*t*) time series were all highly significant (all *p* values <.001; see Table 4). Higher scaling indices were associated with lower errors of commission. Further, the associations were stronger for data in the high versus low time‐stress condition.

**TABLE 3:** Regression analyses of *Y*(*n*) scaling indices predicting errors of commission

Coefficient	Estimate	SE	*t*	*p*	Adj *R* ^2^
Individual sessions					
(Intercept)	0.27	0.21	1.32	.20	
Beta low	−0.14	0.34	−0.42	.68	−0.0293
(Intercept)	0.35	0.30	1.18	.25	
Beta high	0.01	0.48	0.02	.99	−0.0357
All sessions appended					
(Intercept)	0.18	0.06	3.02	.003	
Beta low	0.02	0.10	0.19	.85	−0.00553
(Intercept)	0.35	0.09	3.73	.0003	
Beta high	0.02	0.16	0.11	.92	−0.00585

**TABLE 4:** Regression analyses of *X*(*t*) scaling indices predicting errors of commission

Coeff	Estimate	SE	*t*	*p*	Adj *R* ^2^
Individual sessions					
(Intercept)	1.24	0.17	7.32	8.83 × 10^−12^	
Beta low	−1.41	0.23	−6.20	3.92 × 10*^−^* ^9^	0.177
(Intercept)	2.71	0.28	9.69	6.99 × 10*^−^* ^18^	
Beta high	−3.37	0.40	−8.42	1.67 × 10*^−^* ^14^	0.295
All sessions appended					
(Intercept)	1.34	0.28	4.77	5.19 × 10*^−^* ^5^	
Beta low	−1.53	0.37	−4.11	3.17 × 10^−4^	0.353
(Intercept)	2.96	0.57	5.16	1.81 × 10^−5^	
Beta high	−3.71	0.82	−4.54	9.76 × 10^−5^	0.403

**FIGURE 7:**
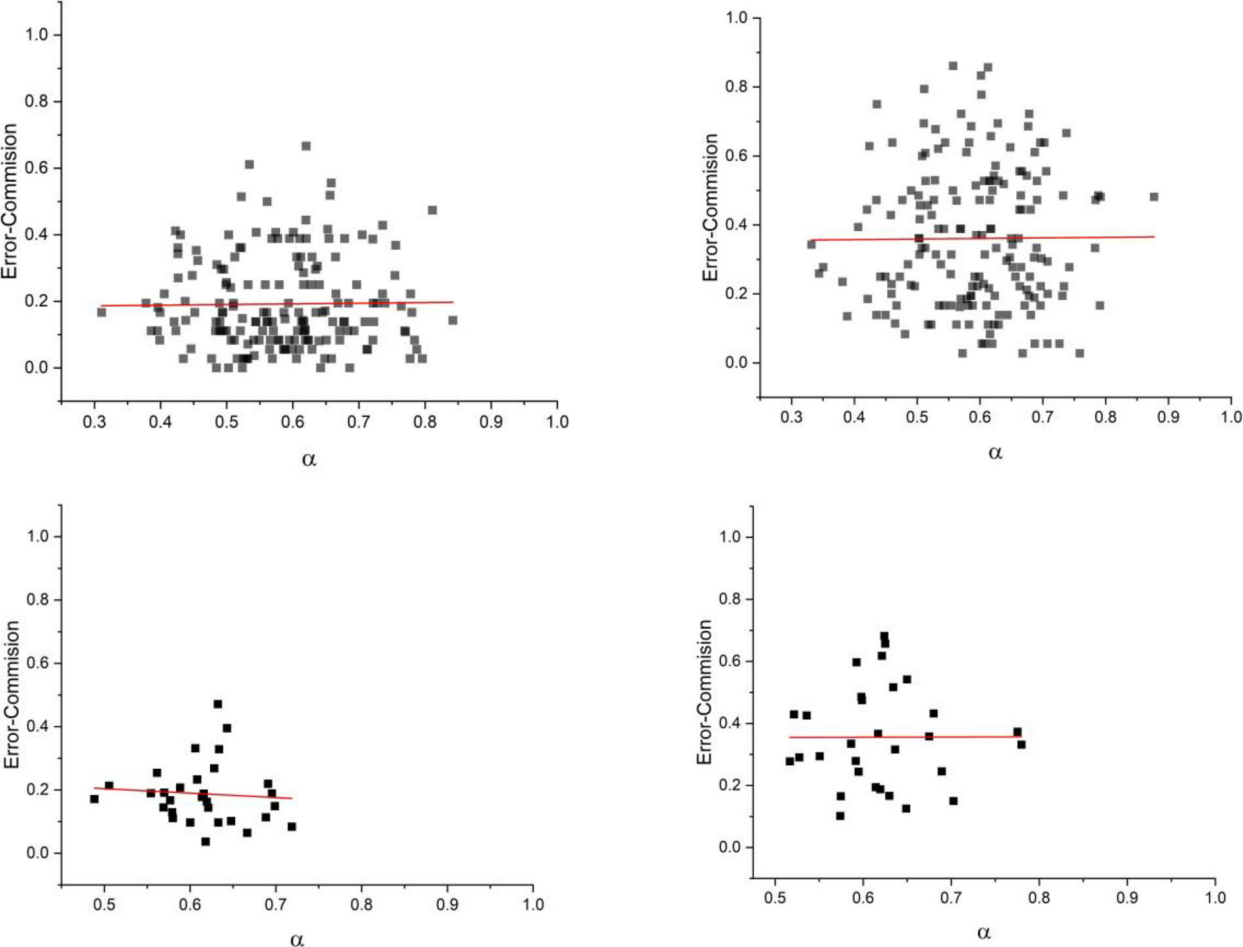
The figures show the probability of errors of commission versus scaling indices of detrended fluctuation analysis (DFA) on *Y*(*n*)’s of all sessions of all participants (top figures) and appended sessions of all the participants (bottom figures) for two cases of low (left figures) and high time‐stress conditions (right figures).

**FIGURE 8:**
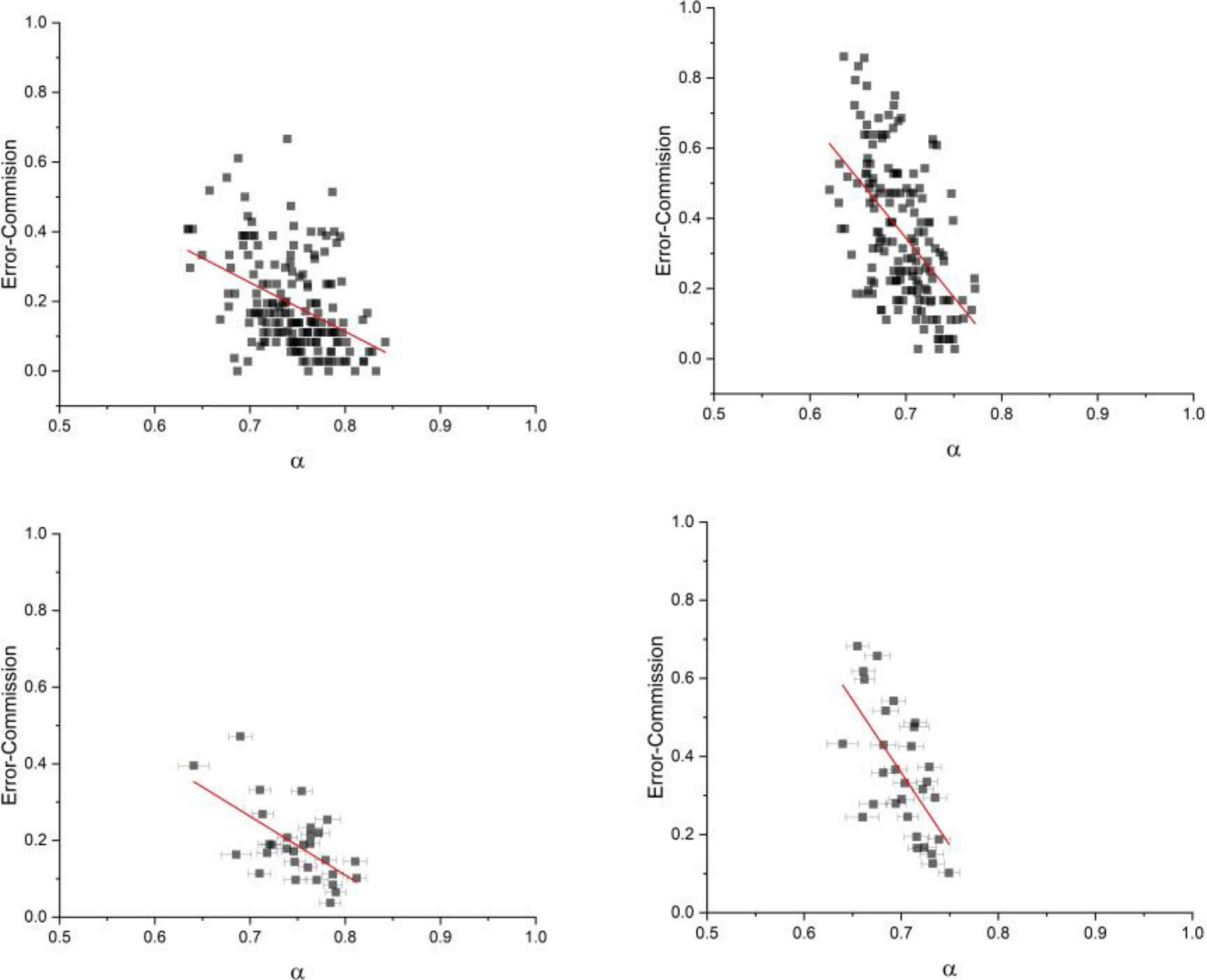
The figures show the trend between the probability of errors of commission and scaling indices of detrended fluctuation analysis (DFA) analysis on the *X*(*t*) time series of all sessions of all participants (top figures) and appended sessions of all the participants (bottom figures) for two cases of low (left figures) and high time‐stress conditions (right figures). Each data point is averaged over 100 DFA on X(*t*)s.

Figures 7 and 8 show the probability of errors of commission versus scaling indices *α*, measured via the DFA processing of *Y*(*n*) and *X*(*t*) datasets, respectively. In each figure, the top and bottom panels correspond to the data of individual sessions and appended sessions, respectively. Moreover, the left panels and right panels are data from low and high time‐stress conditions, respectively. As can be seen, the DFA processing of *Y*(*n*) time series did not reveal any interdependence between the errors of commission of the participants and their value of scaling index (Figure 7). On the other hand, using DFA to process the corresponding *X*(*t*), time series shows a clear trend; participants with higher values of the scaling index have lower errors of commission (Figure 8).

## 4 DISCUSSION

We developed and applied a new approach to measuring the scaling index of reaction time series data using DFA, from a short, complex cognitive‐motor decision‐making task. In this method, we consider each reaction time as a duration time and create a secondary time series *X*(*t*) by filling each time interval with fixed noise of magnitude 1 and a random sign and measured its scaling index using DFA. The *X*(*t*) time series represents the rigidity/flexibility of the process. Using the new method, we were able to show the existence of relations between scaling indices and errors of commission, a measure of cognitive flexibility (consistent with Simola et al., 2017), whereas traditional DFA processing of *Y*(*n*) time series failed to show any such relation. Although Simola et al. (2017) observed IPL scaling of *Y*(*n*) time series from a Go–NoGo task using autocorrelation functions, PSD, and DFA, their task consisted of 1000 equally spaced trials and was presented on a computer monitor requiring keyboard responses. These two methodological differences (lower trial numbers and greater task complexity) may account for why we did not observe the hidden IPL scaling of *Y*(*n*) time series in the present study. Further, the new method of DFA processing of the transformed time series *X*(*t*) was able to discriminate between low and high time‐stress conditions, whereas the DFA analysis of *Y*(*n*) time series was not. Our finding by applying DFA to *X*(*t*) time series is also consistent with previous research, which has observed higher scaling indices in *Y*(*n*) time series associated with lower task demand or decreased task complexity with longer trial series (>1000; Kello et al., 2007) (see also Delignieres et al., 2006).

The stronger negative relation between scaling indices and errors of commission in the high time‐stress condition suggests that under higher time demand conditions (less time to complete tasks), it is beneficial for the system to shift from a more ordered, predictable state to a more disordered, unpredictable state. However, a shift too far in the direction toward disorder results in the deterioration of inhibitory control. Lower scaling indices in the high versus low time‐stress condition suggest that behavior is more disordered or unpredictable (more random) in the high time‐stress condition, an effect that appears to be environmentally coupled to the time constraints imposed by the task. Together, these two findings support previous research suggesting that system complexity is associated with greater degrees of freedom, thus facilitating greater flexibility and adaptability of the system to internal and external perturbations or task demand conditions (Kello et al., 2007; 2010; Van Orden et al., 2003, 2005; Wijnants et al., 2009).

Traditional DFA provides a method to quantify long‐range correlations in time series and to index repeating patterns over different time scales (Peng et al., 1995). As our results reveal, randomly shuffling the *Y*(*n*) time series destroys the temporal structure of the time series, which results in scaling indices of approximately *α* = .5. However, applying DFA to the *X*(*t*) of the shuffled *Y*(*n*) results in scaling indices compatible with those obtained from the original *X*(*t*) time series. This result suggests that the DFA analysis of the *X*(*t*) time series provides a method for detecting CEs (i.e., renewal processes), the time intervals of which between CE are uncorrelated (Turalska & West, 2018) but cannot infer LRTC.

The results of shuffling analysis show that the X(*t*) time series, unlike the traditionally used *Y*(*n*) time series, is insensitive to the order of the reaction times. In our experiment, the time intervals within sessions were randomly distributed and there were longer time intervals between sessions. Due to this property of the method, we could concatenate trials even when temporal distance between trials differed significantly (e.g., between sessions). This made the method suitable for even more realistic experimental and real‐world conditions where intervals between responses to stimuli may vary significantly. In other words, the statistics of *X*(*t*) are renewal, and the time interval between events are statistically independent of one another. Shuffling the *Y*(*n*) will not change the statistics of its corresponding *X*(*t*), so they will again be an IPL with index *μ*.

Now we consider a second time series to which we apply DFA and get a scaling index *λ*. But we know when we shuffle the time series, we are going to obtain a different scaling index, say *α*, because the index *λ* we obtained had to do with long‐range correlations that are destroyed when we shuffle. If the second time series (before shuffling) is fractional Gaussian noise then after shuffling, we would obtain *α* = .5. However, if the time series is mixed, we could obtain *α* = *μ* so that the shuffling acts like a filter, and the deviation of *α* from 0.5 is that part of the mixed time series that is renewal.

We note that the method of subordination to ordinary diffusion proposed by Sokolov (2000) to illustrate the popular theory of continuous time random walk (Scher & Montroll, 1975) is widely adopted to generate the events that are the source of temporal complexity revealed by the statistical analysis of experimental data. For instance, Turalska and West (2018) used this theoretical interpretation to describe the temporal complexity of individuals of a decision‐making social system. Subordination makes it possible to convert the fluctuations of data that would not generate a departure from ordinary diffusion into rare events yielding temporal complexity. Our new method is based on converting the intensity (latency) of reaction times into the time intervals between consecutive events. The time interval between consecutive events is filled with either +1 or −1, tossing a fair coin (see panel c of Figure 2). In Bologna (2020), this approach was used to illustrate the anomalous diffusion properties of events, with a waiting‐time PDF known to be proportional to 1/*τμ* with *τ* denoting the time interval between consecutive events. However, in this paper, the number of reaction times available to us is not large enough to allow us to determine with precision the power law index *μ* from the distribution of the reaction times. We have directly converted the reaction times in the chronological order into a diffusion process without finding a deviation from ordinary scaling. Quite surprisingly we found that the adoption of the current model, used for the first time to the best of our knowledge, to detect the complexity of the experimental fluctuations allows us to evaluate with precision the parameter *μ*.

## 5 CONCLUSIONS

In this study, we introduce a novel conceptual and analytical framework for estimating the complexity of behavioral time series data based on CEs. Analyses of the *X*(*t*) time series, using DFA, we found a clear classification of IPL scaling indices between low and high time‐stress conditions. We also found clear relations between IPL scaling indices and errors of commission. Neither of these findings was observed for the traditional DFA analysis of *Y*(*n*) time series. This new approach was borne out of necessity due to the relatively short reaction time series (360 trials over 8–10 min task durations), which has strong implications for future research in cognitive science and neuroscience experiments, where task durations may be relatively short and where data are collected intermittently within and across recording sessions.

## AUTHOR CONTRIBUTIONS

Scott E. Kerick conceived and conducted the original experiment; Korosh Mahmoodi, Paolo Grigolini, and Bruce J. West conceived and applied the novel DFA analysis. All authors contributed to analyses of results and writing and reviewing the manuscript.

## CONFLICT OF INTEREST STATEMENT

The authors declare no conflict of interests.

### PEER REVIEW

The peer review history for this article is available at https://publons.com/publon/10.1002/brb3.3069.

## Data Availability

The codes and the datasets analyzed during the current study are available at https://github.com/Korosh137/DFA‐RT.git.

## Footnotes

1 Hereafter we replace the term chronological time with event time because the time intervals between reaction time trials during the continuous task are ignored (i.e., time after a response has been made until the time of the next target onset), whereas the durations of the reaction time latencies are taken as events with temporal durations. As mentioned, see Section 2.5 for details of how X(*t*) is obtained from Y(*n*) by means of an analytic transformation.
